# Prevalence of Depression in Pregnant Women with Bariatric Surgery History and Associated Factors

**DOI:** 10.1055/s-0042-1742682

**Published:** 2022-02-25

**Authors:** Andréa Christina Nowak da Rocha, Ana Cristina Barros da Cunha, Jaqueline Ferreira da Silva

**Affiliations:** 1 Professional Master's Program in Perinatal Health, Maternidade Escola da UFRJ (ME-UFRJ), Rio de Janeiro, RJ, Brazil; 2 Professional Master's Program in Perinatal Health, ME-UFRJ; Postgraduate Program in Psychology, Instituto de Psicologia da UFRJ, Rio de Janeiro, RJ, Brazil; 3 Institute of Psychology, UFRJ, Rio de Janeiro, RJ, Brazil

**Keywords:** bariatric surgery, pregnancy, mental health, depression, perinatal care, cirurgia bariátrica, gravidez, saúde mental, depressão, assistência perinatal

## Abstract

**Objective** To analyze the prevalence and factors associated with depressive symptoms among Brazilian pregnant women with history of bariatric surgery (BS).

**Methods** This is a cohort study with 247 women who got pregnant after BS. Based on data collection via Google Form, the recruitment of participants occurred in Facebook groups for 13 months. All of them answered a form with Informed Consent, a general data protocol and the Brazilian version of the Depression, Anxiety and Stress Scale-21. Descriptive and inferential analysis were performed, and a binary logistic regression model was tested to predict the factors associated with depressive symptoms.

**Results** The prevalence of depressive symptoms was 32.8%, noted as being higher in the first (40.6%) and third (34.3%) gestational trimesters. Significative associations were found between depression and marital status ( *p* = 0.000), planned pregnancy ( *p* = 0.001), desired pregnancy ( *p* = 0.004) and psychiatric history ( *p* = 0.000). Women who were not married (odds ratio, OR = 3,38; *p* = 0.002) and had a psychiatric history (OR = 2.70; *p* = 0.102) had higher chances of showing depression symptoms; while planned and desired pregnancy showed as protective factors to the symptoms of depression.

**Conclusion** These findings highlight the importance of psychological assistance for pregnant women with history of BS, to prevent development of mental disorders and their outcomes for maternal-child health.

## Resumo

**Objetivo** Analisar a prevalência e fatores associados à sintomatologia depressiva entre gestantes brasileiras com histórico de cirurgia bariátrica.

**Métodos** Trata-se de um estudo de coorte com 247 mulheres que engravidaram após uma Cirurgia Bariátrica. Baseado em coleta via Google Form o recrutamento das gestantes ocorreu em grupos do Facebook durante 13 meses. Todas responderam a um formulário com o Termo de Consentimento Livre e Esclarecido, um protocolo de dados gerais e a versão brasileira da Depression, Anxiety and Stress Scale-21. Análises, descritiva e inferencial foram realizadas, e um modelo de regressão logística binária foi testado para predizer os fatores associados à sintomatologia depressiva.

**Resultados** A prevalência da sintomatologia depressiva foi de 32,8%, sendo notada como maior no primeiro (40,6%) e terceiro (34,3%) trimestres gestacionais. Associações significativas foram encontradas entre sintomatologia depressiva e estado civil ( *p* = 0,000), gestação planejada ( *p* = 0,001), gestação desejada ( *p* = 0,004) e histórico psiquiátrico ( *p* = 0,000). Mulheres que não eram casadas (OR = 3,38; *p* = 0,002) e tinham um histórico psiquiátrico (OR = 2,70; *p* = 0,102) apresentaram mais chances de manifestarem sintomas de depressão; enquanto gestação planejada e desejada mostraram-se como fatores protetivos aos sintomas de depressão.

**Conclusão** Os achados ressaltam a importância do acompanhamento psicológico para gestantes com histórico de cirurgia bariátrica para prevenção do desenvolvimento de transtornos mentais e seus desfechos para a saúde materno-infantil.

## Introduction

Obesity is a chronic disease which involves genetic, metabolic, behavioral, environmental, cultural, and psychological factors. It is characterized by excess body fat due to energy imbalance for a long period, either by an excessive fat consumption, or by sedentarism or lack of physical activity. 1 The most recent data of Brazilian's Ministry of Health show that 55.7% of the Brazilian population is overweight, in other words, closer to obesity. 2 The rise in obesity in Brazil is justified by the behavioral, social, and dietary transformation of its population. According to Garcia, 3 the urbanization and industrialization binomial is a predominant factor in the way that people eat, since demand for bad and practical eating, as well as fast foods have increased. This demand makes space for higher consumption of invariable foods, without “real food” that can be found as fast food.

Bariatric surgery (BS) has been embraced as an effective option as a preventive and therapeutic intervention to obesity treatment and its related diseases, such as infertility. 4 5 In accordance with the 424/2013 4 decree of Ministry of Health and the new Brazilian Guidelines of Obesity (2016), 5 BS candidates must show a body-mass index (BMI) higher than 40 kg/m ^2^ or 35 kg/m ^2^ , be associated with one or more serious comorbidities caused by obesity, and have attempted, unsuccessfully, non-surgical techniques for losing weight in a prior period of at least two years. 4 5 The greatest benefit of this surgery procedure is the reduction of comorbidities, although it emphasizes gains with regard to self-esteem, sexuality and social interaction among patients. 6

In addition to the search for better health conditions, women who underwent BS can also have the urge to conform with a cultural parameter of slimness, in other words, a female physical ideal. However, the fast physical, nutritional, and psychosocial changes which occur after BS, aside from the newest silhouette (excessive or suddenly thin) may cause psychological disorders, such as depression, anxiety, alcoholism, bulimia, and anorexia, as well as other compulsive behaviors/disorders like compulsive gambling, shopping, and hypersexuality. 6 7

Moreover, it stands out that 80% of the patients who answered the survey were obese women between 18 and 45-years-old. 8 9 In this population, ovarian polycystic syndrome and infertility are usually found, causing more difficulty to become pregnant. 9 Studies reveal the weight loss resulting from BS has a positive impact to female fertility, with better obstetric prognosis and reduction of obstetric complications associated with overweight, such as gestational diabetes, hypertension, preeclampsia, thromboembolism, fetal macrosomia, urinary infection, prematurity, intrauterine fetal death, anesthetic, and surgical complications. 9 10 Nevertheless, women who did BS must be warned about the minimum time to become pregnant after surgery, with the aim to wait for weight stabilization due to deficiency of vitamin, mineral, and protein absorption resulting from digestive physiology changes. 6 9 This time varies from 12 to 18 months, and it corresponds to a minimum hiatus for a post-BS pregnancy with less maternal-fetal risks. 6 9 11

Therefore, the risks arising from physical, psychological, and metabolic alterations caused by BS add up to a high vulnerability to mental disorders during the pregnancy-puerperal cycle, especially on the first and third trimesters of pregnancy, and on 30 days postpartum (puerperium). 12 13 During gestation, this can be explained by issues and fears related to the adjustment period to pregnancy and the proximity to childbirth. 13 14 Besides that, the challenges of the physical and mental transformations related to gestation result in greater emotional ambivalence that must require the women's mental effort to take on a new role as mothers, 15 which can be harder to women with BS history because of the adaptations in physical and mental levels that result from the surgery.

Despite the emphasis given to puerperal psychological disorders such as postpartum depression, mental disorders during pregnancy are not uncommon and have high prevalence, similar to the puerperal period. 16 Depressive episodes are the most frequent during gestation 12 17 and may negatively impact pre- and postnatal care. 12 Depressive mood or anhedonia is one of main symptoms of depression during pregnancy, albeit alterations in sleep and appetite, irritability, loss of libido, psychomotor retardation, and suicidal ideation are also observed and may cause psychosocial impacts on women. 12 Thus, some of these manifestations such as fatigue, alterations in sleeping, appetite, and libido patterns may cause misdiagnosis of depression on pregnancy.

Studies reveal that prevalence rates of depression during pregnancy in developing countries, such as Brazil, are around 20%. 12 17 18 19 20 Among risk factors associated with depression during pregnancy the biggest factor seems to be a history of psychiatric conditions, especially depression. 12 20 Added to that, sociodemographic factors related to poverty, such as unemployment, low income, education, and unwanted pregnancy; and other factors like alcohol dependence and substance abuse, besides lack of social and emotional support are also predictors of depression during pregnancy. 14 18 19 20 It is necessary to stress that the presence of untreated depression during pregnancy can lead to fetal-maternal and obstetric risks like the increase of abortion rates, placenta abnormalities, hemorrhage, prematurity, fetal pain, low selfcare, low weight of the newborn, preeclampsia, and low adherence to prenatal monitoring. 12 16 21 Hence, depression during pregnancy can lead to a higher risk of postnatal depression, has an impact on the mother-child binomial, may compromise the fetal-maternal and mother-child relationship, as well as the child's psychosocial development. 18 22 23

Thus, the early assessment and treatment of depression symptoms during pregnancy is fundamental to reduce negative outcomes to maternal health, fetal development, labor, and the child's health. 12 16 Regarding women who underwent BS, there might be higher risks related to psychological disorders in the face of difficulties of adaptation to physical and mental changes resulted from surgery. The purpose of this research is analyze the prevalence of depressive symptomatology in Brazilian pregnant women with history of BS, and study factors associated with depression incidence, as well as predictive factors of depression in this type of pregnancy.

## Methods

This is a quantitative cohort comprised of 247 pregnant women from different Brazilian regions, which fulfilled the following criteria: age equal or superior to 18 years old, pregnant at the moment of the survey, and who underwent BS before getting pregnant. Women who, despite having a history of BS, answered the survey after childbirth were excluded.

The project was approved by the Comitê de Ética em Pesquisa da Maternidade Escola da UFRJ (CAEE: 65713417.9.0000.5275) before the beginning of data collection, which occurred for 13 months, from October 2017 to November 2018. The study was announced on Facebook groups predominantly formed by women who underwent BS and got pregnant or wanted to get pregnant, for example: Pregnancy after Bariatric Surgery, Gestation after Bariatric Surgery and Being a Mother after Bariatric Surgery. After the research was authorized and the researcher was included by group administrators, the participants were invited to the study through a link with access to a survey elaborated in Google Forms. The survey began with an Informed Consent Form (ICF), which was a necessary condition for the participant to proceed and answer the following data collection tools: 1) general data protocol for socio demographic, psychosocial and clinical data collection (physical and mental health); 2) Brazilian version of the Depression, Anxiety and Stress Scale (DASS-21) 24 to rate the occurrence of depressive symptomatology detected, with a cutoff point of ≥ 14, which would correspond to moderate levels of the disorder for DASS-21.

All social demographic, psychosocial and clinical data (mental and physical health) were processed and analyzed in terms of frequency of occurrence of information collected by the General Data Protocol. Depressive symptomatology data were rated according to DASS-21 scale's instructions. Analyses, inferential and descriptive, were conducted by using Statistical Package for Social Science (SPSS, IBM Corp. Armonk, NY, USA) version 20.0. The Chi-Squared Test was adopted to investigate sociodemographic (marital status, education, labor activity, and familiar income), psychosocial (planned pregnancy, desired pregnancy, emotional support, financial support, and history of obesity) and clinical factors, as well as both physical health (gestational age and time of BS) and mental health (compulsive behaviors and history of psychiatric disorders) associated with depressive symptomatology, assuming *p* -values ≤ 0.05 as statistically significant. Finally, a binary logistic regression model was tested to detect predictive factors of depressive symptomatology in pregnant women with history of BS, controlling the following statistically significant variables ( *p* ≤ 0.05): marital status, planned pregnancy, desired pregnancy, and history of psychiatry disorders.

Finally, it is necessary to emphasize that the variables of history of obesity and compulsive behaviors show as missing because they were inserted after the beginning of data collection. Therefore, these two variables were collected only in 71 participants.

## Results

As can be observed on Table 1 , the sample was mostly from married women (84.6%; n = 209), white women (64.8%; n = 160), between the age of 30 to 34-years-old (38.5%; n = 95), followed by women from 25 to 29-years-old (28.3%; n = 70). More than a half of them had uncompleted or completed college (68.9%; n = 124). Moreover, 74.5% (n = 184) had labor activities, with monthly incomes centered in the group of 2 to 4 minimum wage (43.7%; n = 108), followed by remunerations between 4 to 10 minimum wage (33.2%; n = 82). In the most part, participants lived in the Southeast of Brazil (53.4%; n = 132), and 79.1% (n = 188) of them did prenatal in the private health care system.

**Table 1:** Participants' sociodemographic data (n = 247)

Variables	Category	n	%
**Marital status**	Single	33	13.4
	Married	209	84.6
	Divorced	5	2.0
Total		247	100
**Race/Ethnics**	Yellow	3	1.2
	White	160	64.8
	Mixed race (parda)	61	24.7
	Black	14	5.7
	No answer	9	3.6
Total		247	100
**Age group**	20 to 24 years	18	7.3
	30 to 34 years	95	38.5
	35 to 39 years	54	21.9
	40 to 44 years	10	4
Total		247	100
**Education**	Incomplete elementary school	1	0.4
	Complete elementary school	2	0.8
	Incomplete middle school	8	3.2
	Complete college	116	47.0
Total		247	100
**Labor Activity**	Yes	184	74.5
	No	63	25.5
Total		247	100
**Family income**	No income	7	2.8
	To 2 minimum wage	32	13.0
	From 2 to 4 minimum wage	108	43.7
	From 4 to 10 minimum wage	82	33.2
	From 10 to 20 minimum wage	13	5.3
	More than 20 minimum wage	5	2.0
Total		247	100
**Brazilian region**	North	2	0.8
	Northeast	33	13.4
	Center-West	16	6.5
	Southeast	132	53.4
	South	64	25.9
Total		247	100
**Prenatal assistance**	Public institution	50	20.2
	Private institution	188	76.1
	No monitoring	9	3.6
Total		247	100

In relation to psychosocial data, though half of the women (51%; n = 126) declared unplanned pregnancy, it is understood that gestation was predominantly desired by them (86.6%; n = 214). Nonetheless, 56.3% (n = 139) denied having financial and/or emotional support, 37.7% (n = 93) reported having emotional support, and 3.2% (n = 8) both supports. Most of the women declared being obese since childhood (42.3%; n = 30), followed by adolescence (36.6%; n = 26).

Clinical data of mental and physical health are described in Table 2 . It is noted that 25.9% (n = 64) were found in the first gestational trimester, 34% (n = 84) in the second trimester, and 40.1% (n = 99) in the third trimester. Most participants declared being pregnant after 18 months of BS (63.6%; n = 157) by Gastric Bypass technique (87.4%; n = 216), and are still on medical follow-up (47.4%; n = 117). Most of them (78.9%) reported having some disease before pregnancy, with clinical pathologies such as systemic arterial hypertension, anemia, hypothyroidism, diabetes, musculoskeletal disorders, and hepatic steatosis in more than half of them (53.4%; n = 132); followed by history of psychiatric disorders, such as depression and anxiety/panic attack (12.1%; n = 30), or both (13.4%; n = 33).

**Table 2:** Clinical data about participants' physical and mental health (n = 247)

Indicators of clinical health	Frequency	%
**Gestational age**		
1 to 13 weeks (1 ^st^ Trimester)	64	25.9
14 to 26 weeks (2 ^nd^ Trimester)	84	34.0
27 to 40 weeks (3 ^rd^ Trimester)	99	40.1
Total	247	100
**Time of BS**		
Less than 6 months	7	2.8
6 to 11 months and 30 days	28	11.3
12 to 17 months and 30 days	55	22.3
18 months to 23 months and 30 days	35	14.2
More than 24 months	122	49.4
Total	247	100
**Type of BS**		
Gastric bypass	216	87.4
Vertical gastrectomy	31	12.6
Total	247	100
**Time of medical supervisor after BS**		
Less than 6 months	25	10.1
6 months to 1 years	52	21.1
1 year to 2 years	53	21.5
Still on supervisor	117	47.4
Total	247	100
**Diseases before pregnancy**		
Clinical pathologies	132	53.4
History of psychiatric disorders	30	12.1
Both	33	13.4
None	52	21.1
Total	247	100
**Compulsive behaviors**		
Bulimia nervosa	8	11.3
Anorexia nervosa	2	2.8
Binge eating disorder	13	18.3
Alcohol use disorder	2	2.8
Disorder related to substance abuse (sedatives, hypnotics, anxiolytics or drugs)	1	1.4
Other compulsions (sex, gambling, physical exercise, shopping etc.)	2	2.8
No	43	60.6
Total	71	100
Didn't answer	176	0

Abbreviations: BS, bariatric surgery.

Based on the Diagnostic and Statistical Manual of Mental Disorders (DSM-5) 25 diagnostic criteria: 39.4% (n = 28) of the women declared having compulsive behaviors during pregnancy, with 32.4% (n = 23) being eating compulsions such as bulimia nervosa (11.3%; n = 8), anorexia nervosa (2.8%; n = 2), and binge eating disorder (18.3%; n = 13).

The estimated prevalence of gestational depressive symptomatology of women with history of BS on this survey was of 32.8% (M = 11.4; DP = 11.5; AV = 0–42), with a cutoff point of ≥ 14, which is considered moderate depressive symptomatology level by DASS-21.

On Table 3 were presented the Chi-Squared Test results used to test relations among depressive symptomatology in pregnant women and sociodemographic, psychosocial, and clinical variables. Variables such as marital status ( *p* = 0.000), planned pregnancy ( *p* = 0.001), wanted pregnancy ( *p* = 0.004), and history of psychiatric disorders ( *p* = 0.000) showed statistically significant association with depressive symptoms ( *p* ≤ 0.05). Then, unmarried women, who did not plan or want the pregnancy, and had a history of psychiatric disorders (depression, anxiety, and/or panic attack) had high depressive symptomatology scores identified by DASS-21. Other variables such as education, income, social and emotional support, gestational age, labor activity, history of obesity, time of BS before getting pregnant, gestational trimester, and compulsive behaviors do not show a statistically significant association with depressive scores.

**Table 3:** Data of the relation among depressive symptomatology and participants' sociodemographic, psychosocial and clinical variables (n = 247)

Variables	No depressive symptoms		With depressive symptomatology		
	n	%	n	%	*P* -value
**Marital status**					0.000*
Unmarried	16	41	23	16	
Married	150	72.1	58	150	
**Education**					0.062
Completed and uncompleted elementary school	3	100	0	0	
Completed and uncompleted high school	56	75.7	18	24.3	
Completed and uncompleted college	107	66	55	34	
**Family income**					0.096
No income	4	57.1	3	42.9	
Up to 2 minimum wage	17	53.1	15	46.9	
From 2 to 4 minimum wage	79	73.1	29	26.9	
From 4 to 10 minimum wage	52	63.4	30	36.6	
From 10 to 20 minimum wage	10	76.9	3	23.1	
More than 20 minimum wage	4	80	1	20	
**Labor activity**					0.177
Yes	150	71.1	58	28.9	
No	16	41	23	59	
**Planned pregnancy**					0.001**
Yes	96	77.4	28	96	
No	70	56.9	53	70	
**Desired pregnancy**					0.004*
Yes	151	70.6	63	29.4	
No	15	45.5	18	54.5	
**Emotional support**					0.605
Yes	66	65.3	35	34.7	
No	100	68.5	46	31.5	
**Financial support**					0.238
Yes	8	53.3	7	46.7	
No	158	68.1	74	31.9	
**History of obesity**					0.974
Obese since childhood	21	70	9	30	
Obese as of adolescence	18	69.2	8	30.8	
Obese as of adulthood	10	66.7	5	33.3	
**Gestational age**					0.122
1 ^st^ Trimester	38	59.4	26	40.6	
2 ^nd^ Trimester	63	75	21	25	
3 ^rd^ Trimester	65	65.7	34	34.3	
**History of psychiatric disorders**					0.000*
Yes	31	49.2	32	50.8	
No	45	86.5	7	13.5	
**Compulsive behaviors**					0.197
Eating disorder	11	52.4	10	47.6	
Disorder related to use of some substance	2	66.7	1	33.3	
Other compulsions	1	50	1	50	
No	35	77.8	10	22.2	

Notes: (%) frequency; * *p* ≤ 0,05; **with *p* ≤ 0,001 being statistically significant.

The result of binary logistic regression was significative: X ^2^ (5) = 276.759; *p* < 0.001; R ^2^ Nagelkerke = 0.188, and confirmed that variables like marital status and history of psychiatric disorders were predictors of depressive symptomatology's occurrence in the pregnant women with history of BS participants in this survey. As noted in Table 4 , single women with history of BS proved to be three times (OR = 3.38; 95% CI = 1.586–7.221; *p* = 0.002) more likely to develop depressive symptoms, when compared to married women. Similarly, pregnant women with history of psychiatric disorders showed a more than double chance (OR = 2.70; 95% CI = 1.438–5.081; *p* = 0.102) to develop these symptoms.

**Table 4:** Predictive factors of depressive symptomatology in participants (n = 247)

Variables	β	OR	95% CI	*P* -value
Marital status	1.219	3.38	1.586–7,221	0.002**
Planned pregnancy	-0.530	0.59	0.312–1,111	0.102
Wanted pregnancy	-0.592	0.55	0.234–1,305	0.438
History of psychiatry disorder	0.994	2.70	1.438–5,081	0.002**

Abbreviations: 95% CI, 95% confidence interval; OR, odds ratio. Notes: * *p* ≤ 0,05; **with *p* ≤ 0,001 being statistically significant.

In contrast, planned pregnancy ( *p* = 0.102) and desired pregnancy ( *p* = 0.438), do not perform as significance predictions to depression occurrence in this population. On the contrary, planned pregnancy (β = - 0.530; OR = 0.59) and desired pregnancy (β = –0.592; OR= 0.55) may represent protection factors to this population, since in these cases participants had nearly 40% (41 and 45%, respectively) less probability of having depressive symptoms.

## Discussion

In order to investigate the prevalence and related factors to depressive symptomatology in pregnant women with history of BS, this study shows scientific evidence about the importance of examining depressive symptoms in this population. In accordance with the expected results, women who underwent BS are more likely to have mental disorders during the pregnancy-puerperal cycle, especially in the first and third trimesters of pregnancy. Our results confirmed that the prevalence of depressive symptomatology (32.8%) among pregnant women who underwent BS is higher than in low-risk pregnant women, which is around 20%. 12 17 18 20 Marital status, planned and desired pregnancy, and history of psychiatric disorders are factors that must be considered in prenatal monitoring of this population because they are significantly related to depressive symptoms.

Moreover, it should be noted that this study would be done on newly released Endocrine Technological Disorders Unit in Pregnancy (Unidade de Transtornos Endócrino-Metabólicos na Gestação – UTEM) at the Maternity School of Federal University of Rio de Janeiro, a public service reference center to perinatal health essential care in Rio de Janeiro, Brazil. However, participants' recruitment to the survey in this unit was not possible, bearing in mind the low demand for this service. It must be observed that the adherence of the pregnant population with BS history to public services specialized in gestational health was not frequent. In Rio de Janeiro, in addition to UTEM, only the Public Servants Federal Hospital of the State (Hospital Federal dos Servidores do Estado) offers public prenatal monitoring oriented to post-bariatric pregnant women. In other words, the women who did their prenatal on those services, which mostly focused on low-risk and typical pregnancies, did not receive the necessary care oriented to their characteristics.

Although public health policies in Brazil assure BS indication in cases of severe obesity, like class III, 26 to be adopted, an as a procedure to infertility treatment among women, 27 it was observed that a large part of them sough care in private institutions, where it is not always possible to guarantee a multiprofessional, specialized service, capable of caring for and reducing risks in a pregnancy post-BS. It should also be stressed that UTEM is a unit which aims to create a multiprofessional model of prenatal service to the pregnant population with different types of endocrine metabolic disorders, such as obesity, diabetes, and history of BS. The specialized service to this type of pregnancy is important, due to the demand for attention directed to the related risk factors related to gestation after BS, whose impacts to the mother-child binomial are unquestionable. Studies reveal that these risks have different negative results for the mother, such as nutrient deficiency, 28 that may cause anemia, malnutrition, and intestinal obstruction. 28 And for fetal development, it is observed a higher incidence of congenital anomalies, such as neural tube defect and intrauterine growth restriction, 29 and the consequences to the child's health in their development, such as premature birth and low birth weight. 29

Due to the struggle to gather face-to-face data from UTEM patients, the survey's data collection happened, exclusively, in an online environment. This can, for instance, explain the participants' high level of education. Therefore, the participants were recruited in Facebook groups related to women who got pregnant and/or wanted to get pregnant after BS. In these groups they share their experiences before, during, and after gestation. In this virtual context, we observed that pregnant women seem to adopt various methods to deal with difficulties related to maternity, creating support networks and closer ties with other women in these groups. According to Frossard and Dias, 30 internet groups facilitate communication among peers and enable the gathering, organization, and circulation of information about people's needs, and focus on respective demands. In the groups where the survey was made, a huge circulation of information about various aspects involving a gestation after BS was noted. In the vast majority, the information was practical in nature, without a scientific accuracy and without any commitment to medical discourse. Still, it was possible to note that the participants, for the most part, followed medical advice because they kept up medical monitoring (79.1%) and got pregnant after 18 months of BS (63.3%), which is the minimum period recommended for a low-risk pregnancy 6 9 11 after BS by surgical technique gastric bypass (87.4%), which is, according to Santo et al., 9 the golden pattern to this type of obesity treatment.

Since the research's proposal was to recognize indicators of mental health, more specifically of mood disorders, in pregnant women after BS, we chose to call it “depressive symptomatology” and not “depression”, as depression is a psychopathological disorder which demands wider psychiatric and psychodiagnostic assessments. Likewise, DASS-21 is a scale with good psychometric properties to depression symptoms' measurement in Brazilian population. 24 Although this scale is not validated for use in the pregnant population yet, some cautions were adopted to minimize this limitation of the instrument. To identify pregnant women with depressive symptomatology, higher cutoff points were chosen. Different from cutoff < 9, that indicates there are no depression symptoms in the population in general, the chosen cutoff point was ≥ 14, which would indicate the presence of moderate symptoms in the patients. Here, we clarify that this decision was adopted after doing analyses using two cutoff points: ≥ 10, which identifies mild symptoms, and ≥ 14, for moderate symptoms. the first cutoff found a prevalence of 46.9%, which is higher than the prevalence of depression among low-risk pregnant women (20%). 12 17 18 20 Therefore, the cutoff ≥ 14 would be more indicated to investigate the presence of depression symptoms in the population observed, even without a support of DASS-21's psychometric study of sensitivity and specificity for use in pregnant women.

Thus, it is important to discuss how the prevalence of 32.8% depressive symptomatology in pregnant women after BS was higher when compared to that in low-risk pregnant women. 12 17 18 Depression symptoms were more frequent in the first (40.6%) and third trimesters (34.3%), which reinforces the need for policies focused on pregnancy health in the most critical moments during gestation, either by issues related to pregnancy adaptation or to the fear of childbirth. 13 14 This response reinforces the idea that gestation is, indeed, a difficult and vulnerable time in a woman's life which can lead to psychiatric disorders 12 13 particularly associated with physical, hormonal, mental, and social changes, specific from this period. 8 16

Regarding the support system, although most of the participants were married (84.6%), 56.3% denied having any type of financial and/or emotional support. Our data indicates that single women with history of BS showed three times more chances (OR = 3.38; 95% CI = 1.586–7.221; *p* = 0.002) to manifest depressive symptoms, when compared with married women, which highlights the importance of support systems (emotional and social) to face the issues and insecurities that the pregnancy-puerperal period can bring.

Likewise, the presence of psychiatric history (anxiety, panic attack, and depression) found in 25.5% of the participants must be considered, since psychiatric disorders previous to pregnancy may have obstetric, neonatal, and puerperal impacts. The increase of abortion rates, 12 16 18 hemorrage, 12 16 18 low birth weight, 12 16 18 and the consequences to psychosocial child development, 12 16 18 are examples of negative outcomes to the mother-child binomial related with these disorders during the pregnancy-puerperal cycle. Moreover, psychiatric backgrounds may result in a lesser adherence to prenatal care, 12 16 18 with difficulties for the woman to adopt self-care habits, 16 18 impacts to the quality of the maternal-fetal relationship during pregnancy, and the mother-child relationship on puerperium, especially for its association with postnatal depression. 12 16 18 Ratified these risks, we verified that the participants with psychiatric backgrounds demonstrate double chances (OR = 2.70; 95% CI = 1.438–5.081) of manifesting depression symptoms on gestation after BS.

These findings deserve attention because 39.4% of the participants also report compulsive behaviors, especially disorders related to eating, such as binge eating disorder (18.3%), bulimia nervosa (11.3%), and anorexia nervosa (2.8%). Studies suggest these behaviors are common in patients with history of BS 6 7 29 31 32 and may indicate the destination of voracity, despair, and eating impulse 31 32 in these pregnant women. Paradoxically, these behaviors were not related to depressive symptomatology in the participants of this study. Although BS has weight loss as objective, as well as mental and physical health improvement, the presence and/or conservation of compulsive behaviors in pregnant women seem to work as a strategy to relieve their anguish, with effects on mood responses such as depression during pregnancy. We may assume that compulsive behaviors involved in this period can be moderators of the anxiety typical during pregnancy, and high prevalence of depressive symptomatology during pregnancy after BS.

Despite the improvement of metabolic and reproductive functioning after the surgery procedure, 5 9 11 the decision to do a BS does not seem to be a determinant to the intent of getting pregnant, as there was no significative difference between the women who planned (49%) and those who did not (51%) their pregnancies. Nevertheless, most of them (86.6%) affirmed that the gestation was desired. It should be reinforced that neither planned pregnancy (β = -0.53; OR = 0.58; *p* = 0.102) nor desired pregnancy (β = -0.59; OR = 0.55; *p* = 0,438) were predictive factors for depressive symptoms. On the contrary, these factors appear as protectives to the occurrence of depression symptoms for this population, since women who planned and wanted to get pregnant had around 40% (41 and 44% respectively) less chances to manifest those symptoms. This suggests that pregnancy planning and willingness are protective factors to the mental health of pregnant people with a history of BS.

Most of the women affirmed that they were obese since childhood (42.3%) or adolescence (36.6%). Although there is no significative association with depression indicators, we may infer that, despite weight gain before pregnancy, the pressure for a perfect body reduces during gestation due to the expected physical transformations 28 and, possibly, there is a sort of social tolerance for the pregnant body to be a nonstandard body imposed by thinness culture. In spite of that, although gestational age is not associated significantly with depression symptoms, these symptoms were more frequent in pregnant women in the first (40.6%; n = 26) and third trimesters (34.3%; n = 34). It seems to indicate that, as literature has already considered, there is a variation between risks for mental disorders during the pregnancy-puerperal cycle, 12 13 when the second trimester is a moment of emotional stability. Presumably, the difficulties during the adaptation to physical, mental, and social transformations, typical from pregnancy after BS, may result in a higher emotional ambivalence that require women's mental efforts to take a new role as mother. 15 And, still, it seems that due to the physical transformations during gestational trimesters, with the changes of a pregnant body and the alterations of body image, women are more vulnerable to the manifestation of depressive symptomatology, even if it is not significantly related to history of obesity.

Finally, some limitations of this study must be indicated. First, while online collection via Facebook groups enabled this research and expanded its reach, by recruiting participants from different parts of Brazil, the lack of face-to-face meetings between researcher and participants may have caused difficulties to clarify doubts about the survey. Although this strategy of online survey is becoming more disseminated, especially during the Coronavirus pandemic, this type of data collection precludes capture of nuances which face-to-face collection of data may generate, although the participants might feel more comfortable answering the study online. A second important limitation involves the DASS-21 scale used to identify participants with depression symptoms. We reinforce that although it might be a limitation to this study, the choice of DASS-21 was due to it being an easy instrument of application and analysis, besides having a user-friendly language, mainly by collecting data in a virtual environment. For this reason, the studies suggest we use more tools with the purpose of expanding scientific knowledge and the recognition of risk factors and protection related to this type of gestation.

In conclusion, the evaluation of possible mental disorders during pregnancy with history of BS is fundamental, since identifying the risk factors and protections associated with these cases would allow the medical staff to plan specific and preventive interventions for the negative outcomes that this condition imposes. The discoveries in this study ratify the importance of early tracking, diagnostic, and treatment to reduce the impact of perinatal mental disorders, both for the mother and child's health. 12 20

## Conclusion

Therefore, we concluded that the prevalence of depressive symptomatology in pregnant women with BS is higher than what was found in low-risk pregnancies, proving the psychological control of these women is related to prenatal factors, since this condition may show impacts to the mother-child binomial with short, medium and long-term outcomes. Some factors, such as marital status, planned pregnancy, desired pregnancy, and psychiatric history must be observed by the professionals involved. Henceforth, it is necessary to emphasize that planned and desired pregnancies seem to succeed as protection factors to depressive symptomatology, even if these topics were poorly studied into the perinatal psychology field. Considering obstetric mental disorders, serious consequences to fetal-maternal health, and the underdiagnosis of these disorders in this period, the results of this research indicate the importance of psychological support to pregnant women who underwent BS. Moreover, the training of multiprofessional teams is fundamental for the early recognition and strategic orientation for pregnant women with some symptoms of those disorders, for the sake of a better psychodiagnostic assessment and appropriate treatment, minimizing the negative impacts on obstetric and neonatal care. This study highlights the importance of new studies about depression in pregnancy, so that they can subsidize clinical eye development, sensitive and wide, which is oriented to subjective questions from pregnant women with BS, bearing in mind a total attention to perinatal health care of these women and their families.
